# Recovery of Chronic Inflammatory Demyelinating Polyneuropathy on Treatment With Ocrelizumab in a Patient With Co-Existing Multiple Sclerosis

**DOI:** 10.1177/11795735221084837

**Published:** 2022-03-28

**Authors:** Michael Auer, Harald Hegen, Anna Hotter, Wolfgang Löscher, Klaus Berek, Anne Zinganell, Elena Fava, Paul Rhomberg, Florian Deisenhammer, Franziska Di Pauli

**Affiliations:** 1 Department of Neurology, Medical University of Innsbruck, Innsbruck, Austria; 2 Department of Neuroradiology, Medical University of Innsbruck, Innsbruck, Austria

**Keywords:** ocrelizumab, chronic inflammatory demyelinating polyneuropathy, multiple sclerosis, treatment, off-label, anti-CD20

## Abstract

The chimeric anti-CD20 antibody rituximab has demonstrated good efficacy as an off-label treatment in chronic inflammatory demyelinating polyneuropathy (CIDP), while the humanized anti-CD20 antibody ocrelizumab has been approved for treatment of multiple sclerosis (MS), whereas there is no evidence for its use in CIDP so far. We present a patient suffering from CIDP and MS, both refractory to standard treatment and both showing marked improvement on ocrelizumab. To the best of our knowledge, this is a unique report of CIDP with an almost full electrophysiological recovery on ocrelizumab which could be considered as a potential treatment option for refractory CIDP.

## Background

Chronic inflammatory demyelinating polyneuropathy (CIDP) is a chronic autoimmune inflammatory disorder of the peripheral nervous system. First-line treatments of CIDP include intravenous immunoglobulins (IVIg), corticosteroids, and plasmapheresis. Beside these standard treatments several off-label immunomodulatory or immunosuppressive treatments are used particularly in treatment-refractory disease.^1^ One of these off-label treatment options which has shown good efficacy is rituximab, an anti-CD20 antibody.^2^ The humanized or full human anti-CD20 antibodies ocrelizumab and ofatumumab have been approved for treatment of other diseases such as multiple sclerosis (MS), however these biologicals have not been regularly used for treatment of CIDP so far. In MS, a chronic demyelinating autoimmune disease of the central nervous system, ocrelizumab is a highly effective therapeutic option especially in highly active disease courses.^3^ Here, we present the case of a patient with co-existing MS and CIDP who was treated with ocrelizumab which led not only to freedom of MS disease activity but also to nearly full recovery of CIDP previously fluctuating over years. To our knowledge, there is only one reported case of CIDP successfully treated with ocrelizumab.^4^

Written informed consent for patient information and images to be published was provided by the patient.

## Case Report

In October 2017, 2 weeks after a respiratory tract infection, a 26 year old, otherwise healthy, male patient experienced slowly progressive, at the beginning slightly asymmetric, left-side dominant ascending sensory and motor deficits first in both legs, then also affecting both hands. When he was not able to walk longer distances anymore and distal and proximal motor and sensory deficits were present in all four limbs, he was admitted to a hospital in his home country in January 2018. Nerve conduction studies showed typical non-uniform features of demyelinating and motor-dominant neuropathy leading to diagnosis of CIDP according to the accepted and recently revised diagnostic criteria.^5^ The patient was initially treated with plasmapheresis and intravenous immunoglobulins (IVIg), followed by a long-term therapy with oral corticosteroids due to poor response on IVIg. Under this treatment, motor deficits improved; however, he complained of persisting fluctuating sensory symptoms in both legs.

In August 2018, the patient developed an impaired vision on his left eye. Diagnostic work-up including visual evoked potentials showed optic neuritis and typical radiologic findings of a multiple sclerosis (MS) with demyelinating white matter lesions disseminated in space and time. After migration to Austria, he presented the first time at Innsbruck Medical University in July 2019 with an acute optic neuritis on the right eye. Both diagnoses, MS (according to the revised McDonald criteria 2017^6^) and CIDP, were confirmed by clinical, radiological (multiple cerebral and spinal demyelinating lesions) and neurographical (demyelinating neuropathy) findings. Antibodies against nodal and paranodal proteins (Neurofascin 155, Contactin 1, CASPR1), well characterized onconeural antibodies (Yo, Hu, Ri, Ma2, CV2, Amphiphysin) as well as antibodies against PKCγ, CARPVIIII, ARHGAP26, SOX1, GAD65, AK5 and Homer3 were not detected. MOG-IgG and AQP4 antibodies were negative as well. Cerebrospinal fluid work-up showed normal findings at that time. Neurologic symptoms improved after high-dose intravenous methylprednisolone (HDMP). Because MS was the clinically leading disorder at that time with 2 relapses within 1 year, treatment with dimethylfumarate (DMF) was started in August 2019.

**Figure 1.**
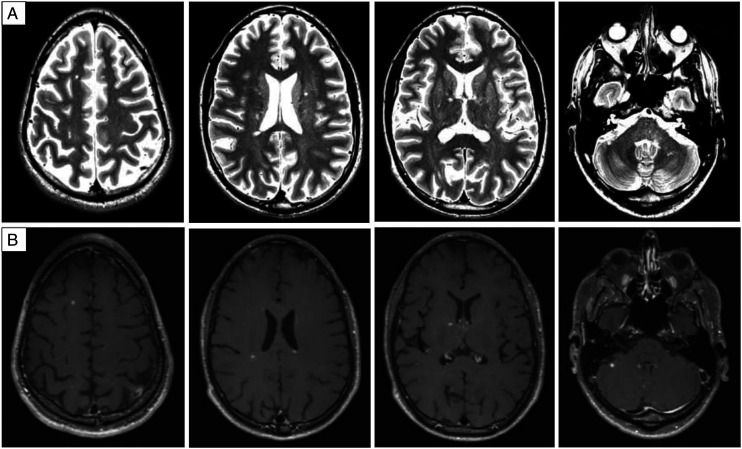
Brain MRI before start of ocrelizumab. April 24, 2020. Brain MRI after multiple sclerosis relapse and high-dose intravenous methylprednisolone therapy, while on treatment with dimethylfumarate, revealed multiple T2 hyperintense white matter lesions, partially in typical areas for multiple sclerosis (A: T2-weighted images) and several of them with contrast enhancement (B: T1-weigted images with gadolinium). While this figure shows only some representative slices, the examination demonstrated 15 new lesions compared to the previous MRI nine months earlier.

However, in December 2019 the patient was admitted again with progressing, symmetric, sensorimotor deficiency of all four extremities. Tendon reflexes were absent and nerve conduction studies showed a marked worsening of CIDP with missing F-waves of the median nerve, longer F-wave latency of the ulnar and tibial nerve with A-waves, temporal dispersion and conduction blocks, as well as reduced sensory amplitudes of the ulnar and median nerve. Therefore, IVIg 2 g/kg was administered which led to incomplete regression of the neurologic symptoms. In February 2020, the patient was given IVIg (1 g/kg) again while MS treatment with DMF was continued. In March 2020 the patient presented with multiple new neurologic symptoms such as diplopia, mild gait ataxia and worsening of sensory deficits in all extremities (Expanded Disability Status Scale, EDSS, 2.5). Because worsening of both, MS (brain stem and cerebellar symptoms) and CIDP (sensory symptoms) occurred, HDMP in a total dosage of 3 g was administered with only partial improvement. MRI showed several new and contrast-enhancing white matter lesions (Figure 1) while electrophysiological findings regarding CIDP remained unchanged with obvious signs of an immune-mediated polyneuropathy (see Table 1). Because MS and CIDP were not sufficiently treated at that time treatment was switched to ocrelizumab in June 2020, while he experienced another optic neuritis few days before start of ocrelizumab. By December 2020, however, the patient reported full recovery of all symptoms showing a normal neurologic examination (EDSS 0) except for absent reflexes due to CIDP. Normal examination with an EDSS of 0 and no reported symptoms regarding CIDP were sustained in April 2021. Nerve conduction studies of median and ulnar nerve confirmed an impressive improvement with almost normalized sensory and motor amplitudes, F-wave latencies and nerve conduction velocities (Table 1).

**Table 1.** Neurographical findings before (March 31, 2020) and 10 months after (April 29, 2021) first infusion of ocrelizumab

	March 2020	April 2021	Normal reference values
Right median nerve			
SCV wrist-dig II [m/s]	Missing	51.7	>45
SCV elbow–wrist [m/s]	Missing	29.6	>45
Sensory amplitude wrist-dig II [μV]	Missing	16.3	>10
Sensory amplitude elbow-dig II [μV]	Missing	9.7	>10
Motor latency wrist-APB [ms]	4.39	3.21	<4.2
MCV elbow–wrist [m/s]	29.8	41.7	>49
Motor amplitude wrist-APB [mV]	3.5	8.6	>5
Motor amplitude elbow-APB [mV]	2.9	7.3	>5
F-wave latency min [ms]	F missing, A waves	30.8	<31
Right ulnar nerve			
SCV Wrist-dig V [m/s]	53.6	47.9	>45
Sensory amplitude wrist-dig V [μV]	5.4	24.2	>8
Motor latency wrist-ADM [ms]	3.08	2.50	<3.3
MCV bl. elbow–wrist [m/s]	41.1	43.4	>50
MCV ab. elbow-bl. elbow [m/s]	36.2	40.4	>50
Motor amplitude wrist-ADM [mV]	4.1	6.6	>4
Motor amplitude bl. elbow-ADM [mV]	2.0	4.6	>4
Motor amplitude ab. elbow-ADM [mV]	1.46	4.6	>4
F-wave latency min [ms]	74.4, A waves	33.9	<30

Abbreviations: ab, above; ADM, abductor digiti minimi; APB, abductor pollicis brevis; bl, below; dig, finger; MCV, motor conduction velocity; SCV, sensory conduction velocity.

Neurographical findings before and after treatment start with ocrelizumab, exemplary shown in detail for the right median and ulnar nerve. Improvement or even normalization of motor and sensory amplitudes, motor latency, motor and sensory conduction velocity, and F-wave latency of both nerves is demonstrated. Compound muscle action potentials were measured from peak to base.

## Discussion

MS and CIDP are both autoimmune, demyelinating neurological diseases. Co-occurrence of both diseases is uncommon but has been reported earlier. The condition of demyelination present in the central and peripheral nervous system is also known as combined central and peripheral demyelinating disease (CCPD) with a large heterogeneity of clinical presentation and disease course.^7,8^ One case of successful treatment of CCPD with rituximab has been published so far.^9^ Contrary to most case reports about CCPD our patient did not simultaneously develop peripheral and central demyelination so that both CIDP and MS were independently diagnosed at different time points. Both, the diagnostic criteria for CIDP^5^ and MS^6^ were fulfilled in this case. Long-term treatment strategies in MS and CIDP are fundamentally different,^10^ however, the anti-CD20 antibody rituximab has been used as an off-label treatment in both diseases. The efficacy of rituximab has particularly been reported in patients with demyelinating polyneuropathy associated with antibodies against paranodal proteins^11^ and in patients with the MADSAM (multifocal acquired demyelinating sensory and motor neuropathy) subtype,^12^ whereby the pathomechanism in these patients may be different to typical CIDP with macrophage-induced demyelination.^13^ While ocrelizumab has been approved for treatment of active relapsing-remitting (and primary progressive) MS there is no evidence for its use in CIDP. Recently, one case has been published with ocrelizumab having been successfully used in a patient with CIDP refractory to standard treatments who developed anti-rituximab antibodies.^4^ The authors were able to show improvement of neurophysiological findings following initiation of ocrelizumab. Our patient suffered from both MS and CIDP, whereby both diseases were not sufficiently controlled under standard treatments. There are no guidelines as to how to treat patients in this special situation. By introducing ocrelizumab as a potential treatment option for both diseases we observed a stabilization of MS disease course with no further relapses and substantial improvement of CIDP. Clinical symptoms related to CIDP and previously present over several years resolved completely and electrophysiological findings markedly improved during ongoing treatment with ocrelizumab.

## Implications for Clinical Practice

We suggest that ocrelizumab could be considered as off-label treatment for CIDP, also due to lower immunogenicity than that of rituximab (26–37% anti-drug antibodies against rituximab compared to .4% against ocrelizumab),^3,14^ and in the rare case of co-occurrence with MS, it seems suitable as treatment of both diseases.

## References

[1] Reynolds J Sachs G Stavros K . Chronic inflammatory demyelinating polyradiculoneuropathy (CIDP): clinical features, diagnosis, and current treatment strategies. R I Med J. 2016;99(12):32-35. 27902997

[2] Muley SA Jacobsen B Parry G , et al. Rituximab in refractory chronic inflammatory demyelinating polyneuropathy. Muscle Nerve. 2020;61(5):575-579. doi:10.1002/mus.26804. 31922613

[3] Hauser SL Bar-Or A Comi G , et al. OPERA I and OPERA II clinical investigators. ocrelizumab versus interferon beta-1a in relapsing multiple sclerosis. N Engl J Med. 2017;376(3):221-234. doi:10.1056/NEJMoa1601277. 28002679

[4] Casertano S Signoriello E Rossi F , et al. Ocrelizumab in a case of refractory chronic inflammatory demyelinating polyneuropathy with anti-rituximab antibodies. Eur J Neurol. 2020;27(12):2673-2675. doi:10.1111/ene.14498. 32875645

[5] Van den Bergh PYK Doorn PA Hadden RDM , et al. European academy of neurology/peripheral nerve society guideline on diagnosis and treatment of chronic inflammatory demyelinating polyradiculoneuropathy: report of a joint task force-second revision. Eur J Neurol. 2021;28(11):3556-3583. doi:10.1111/ene.14959. 34327760

[6] Thompson AJ Banwell BL Barkhof F , et al. Diagnosis of multiple sclerosis: 2017 revisions of the McDonald criteria. Lancet Neurol. 2018;17(2):162-173. doi:10.1016/S1474-4422(17)30470-2. 29275977

[7] Suanprasert N Taylor BV Klein CJ , et al. Polyneuropathies and chronic inflammatory demyelinating polyradiculoneuropathy in multiple sclerosis. Mult Scler Relat Disord. 2019;30:284-290. doi:10.1016/j.msard.2019.02.026. 30870805

[8] Cortese A Franciotta D Alfonsi E , et al. Combined central and peripheral demyelination: clinical features, diagnostic findings, and treatment. J Neurol Sci. 2016;363:182-187. doi:10.1016/j.jns.2016.02.022. 27000248

[9] Makkawi S Yonbawi F Qari Y Aljinaid M . Combined central and peripheral demyelinating disease with good response to B-cell depleting therapy. Cureus. 2021;13(4):e14690. doi:10.7759/cureus.14690. 34055534 PMC8153962

[10] Melzer N Meuth SG . Disease-modifying therapy in multiple sclerosis and chronic inflammatory demyelinating polyradiculoneuropathy: common and divergent current and future strategies. Clin Exp Immunol. 2014;175(3):359-372. doi:10.1111/cei.12195. 24032475 PMC3927897

[11] Querol L Rojas-García R Diaz-Manera J , et al. Rituximab in treatment-resistant CIDP with antibodies against paranodal proteins. Neurol Neuroimmunol Neuroinflamm. 2015;2(5):e149. doi:10.1212/NXI.0000000000000149. 26401517 PMC4561230

[12] Motte J Fisse AL Köse N , et al. Treatment response to cyclophosphamide, rituximab, and bortezomib in chronic immune-mediated sensorimotor neuropathies: a retrospective cohort study. Ther Adv Neurol Disord. 2021;14:1756286421999631. doi:10.1177/1756286421999631. 33747132 PMC7940507

[13] Koike H Kadoya M Kaida K-i , et al. Paranodal dissection in chronic inflammatory demyelinating polyneuropathy with anti-neurofascin-155 and anti-contactin-1 antibodies. J Neurol Neurosurg Psychiatry. 2017;88(6):465-473. doi:10.1136/jnnp-2016-314895. 28073817

[14] Dunn N Juto A Ryner M , et al. Rituximab in multiple sclerosis: frequency and clinical relevance of anti-drug antibodies. Mult Scler. 2018;24:1224-1233. doi:10.1177/1352458517720044. 28762877

